# Methods for Measuring and Estimating Methane Emission from Ruminants

**DOI:** 10.3390/ani2020160

**Published:** 2012-04-13

**Authors:** Ida M. L. D. Storm, Anne Louise F. Hellwing, Nicolaj I. Nielsen, Jørgen Madsen

**Affiliations:** 1 Department of Large Animal Sciences, University of Copenhagen, Grønnegårdsvej 2, DK-1870 Frederiksberg C, Denmark; E-Mail: imld@life.ku.dk; 2 Department of Animal Science, Aarhus University, AU-Foulum, P.O. Box 50, DK-8830 Tjele, Denmark; E-Mail: AnneLouise.Helwing@agrsci.dk; 3 AgroTech, Agro Food Park 15, DK-8200 Aarhus N, Denmark; E-Mail: ncn@agrotech.dk

**Keywords:** methane, ruminants, estimation methods, limitations

## Abstract

**Simple Summary:**

Knowledge about methods used in quantification of greenhouse gasses is currently needed due to international commitments to reduce the emissions. In the agricultural sector one important task is to reduce enteric methane emissions from ruminants. Different methods for quantifying these emissions are presently being used and others are under development, all with different conditions for application. For scientist and other persons working with the topic it is very important to understand the advantages and disadvantage of the different methods in use. This paper gives a brief introduction to existing methods but also a description of newer methods and model-based techniques.

**Abstract:**

This paper is a brief introduction to the different methods used to quantify the enteric methane emission from ruminants. A thorough knowledge of the advantages and disadvantages of these methods is very important in order to plan experiments, understand and interpret experimental results, and compare them with other studies. The aim of the paper is to describe the principles, advantages and disadvantages of different methods used to quantify the enteric methane emission from ruminants. The best-known methods: Chambers/respiration chambers, SF_6_ technique and *in vitro* gas production technique and the newer CO_2_ methods are described. Model estimations, which are used to calculate national budget and single cow enteric emission from intake and diet composition, are also discussed. Other methods under development such as the micrometeorological technique, combined feeder and CH_4_ analyzer and proxy methods are briefly mentioned. Methods of choice for estimating enteric methane emission depend on aim, equipment, knowledge, time and money available, but interpretation of results obtained with a given method can be improved if knowledge about the disadvantages and advantages are used in the planning of experiments.

## 1. Introduction

Livestock and mainly ruminants account for up to one third of the emitted methane worldwide [1], and methane has a greenhouse potential 25 times that of CO_2_ [2]. Therefore methane accounts for a great part of the emitted CO_2_-equivalents from agriculture. Over the last 100 years several different methods have been developed with the purpose of measuring and estimating methane emissions from ruminants. These methods have various scopes for application, advantages and disadvantages—but none of them are perfect: Some are expensive, some cheaper; some suited for grazing animals, some for housed livestock; some can handle many animals, some only few. This all affects the measuring results and our interpretation of them. It is therefore important to know the possibilities and limitations of each method. This applies to the understanding of current research results and to the planning of future projects. The present literature within techniques for estimating greenhouse gas emissions from livestock is primarily concerned with individual methods and their validation. Apart from a review with emphasis on grazing livestock was published in 2007 [3], literature comparing a range of different estimation approaches is scarce.

This review briefly presents the most common methods for estimating and measuring methane emissions from ruminants, including newly developed techniques. The focus is on methods at the individual animal scale. Each method is presented and advantages and disadvantages emphasized. Finally, the descriptions are summarized to facilitate comparison.

## 2. Measuring Methane by Means of Chambers

Different chamber systems or respiration chambers have been used for the last 100 years with the main purpose of studying the energy metabolism of animals [4,5]. Methane loss is an inherent part of the energy metabolism in ruminants, and various types of chambers are valuable tools in the investigation of mitigation strategies for methane emissions.

**Figure 1:**
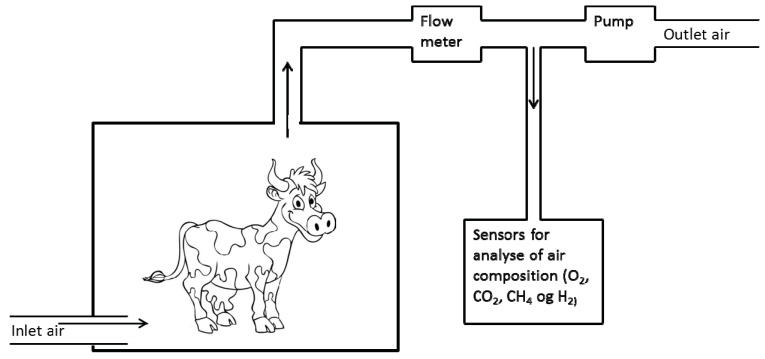
Diagram of open circuit respiration chamber.

The principle of the chambers is to collect all exhaled breath from the animal and measure e.g., the methane concentration. Animal calorimetric systems, where air composition is measured, are divided into two main types: The closed-circuit [6] and the open-circuit, with the latter being the dominating one [5]. In Figure 1 an outline of an open-circuit system is shown. A pump pumps air from the chamber through a flow meter and different gas sensors. Fresh air for the animal is drawn from outside. In some systems fresh air is drawn through an air conditioning system to control humidity, temperature and mixing of air in the chamber but air can also simply be taken from outside the chamber. The methane emission is calculated from flow and gas concentration in inlet and outlet air from the chamber, but more complex calculations have been developed that also take into account the small differences in inflow and outflow and changes in chamber concentration of gases [7]. The difference between the outgoing and incoming amount of methane corresponds to the methane emission. Many different chambers have been constructed on the basis of this principle including insulated chambers with controlled temperature and humidity [5,8,9,10], more simple types with no insulation of chambers and fresh air inlet from the room [11,12,13], systems where just the head of the animal is placed in the chamber [14,15,16] and systems developed to measure grazing animals [17,18,19].

Chambers are regarded as the standard method for estimation of methane emission from ruminants, because the environment can be controlled and the reliability and stability of instruments can be measured [5,20]. However, there is a risk of creating an artificial environment, which affects animal behavior e.g., dry matter intake (DMI). As DMI is one of the main drivers of methane emission a decrease in DMI would not only effect total emission but also the derived estimates like loss of gross energy [21]. Therefore, it has been queried that results obtained in chambers cannot be applied to free ranging animals e.g., animals on pasture [22,23]. Investigations have shown that chambers give more precise estimates of methane emissions than the SF_6_ tracer technique [11].

**Figure 2:**
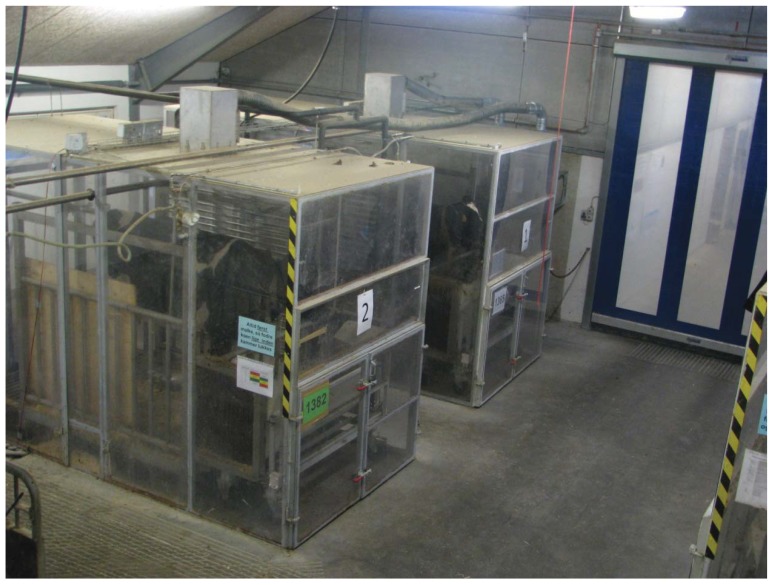
Respiration chambers constructed at Aarhus University of a steel frame covered with polycarbonate.

Classical chambers for energy metabolism with air conditioning, internal mixing of air and careful tightening to reduce the risk of air loss to the surroundings [8] are expensive to build. Therefore less expensive systems have been developed with methane measurements as the main purpose [11,12,13]. In Denmark four chambers based on open circuit calorimetry have been built. The chambers are 1.8 m (witdh) × 2.5 m (height) × 3.8 m (length) with a volume of approximately 17 m³. The chambers are constructed of a metal frame covered with transparent polycarbonate walls (Figure 2). They are placed so cows can have visual contact with other animals in an existing barn to ensure animal welfare and dry matter intake. Air is drawn from the barn and concentrations of CH_4_, O_2_, CO_2_, and H_2_ are measured in inlet and outlet air. DMI measured before and during chamber stays have shown that feed intake is unaltered (Hellwing, unpublished data). It clearly shows that design and placement of chambers can reduce the risk of creating an artificial environment and eliminate the risk of reduced DMI. Furthermore, data on methane emission can be combined with data on rumen metabolism and digestibility [24], increasing our understanding of methane production and metabolism.

Nearly all aspects of feeding and nutrition can be investigated in a chamber system. The level of feeding, effect of feedstuff, effect of chemical and physical composition, restricted *versus* *ad libitum* feeding, different feeding schedules, different additives *etc*. Also changes in emission during the day can be described with the system, but resolution will depend on the number of measurements during a given day.

The variation in measurements is affected by instrument variation as well as within and between animal variations. Within animal variation or day-to-day variation will affect the number of days needed for measuring. The day-to-day coefficient of variation (CV) has been reported to be 7.2% in cattle and sheep [25], 4.3% for dairy cows [26] and 4.7% in sheep [11]. Increasing the number of measuring days will decrease the random error [27]. For methane or energy metabolism studies of three to five days has been used [11,25,26]. The between animal CV has been reported to be 7–8% in restricted fed sheep [25], 17.8% for *ad libitum* fed cows [26], and 6.1% in restrictively fed sheep [11]. A higher between animal variation during *ad libitum* feeding than during restricted feeding corresponds well with the findings of Thorbek [28] who studied CO_2_ and CH_4_ production by growing calves at high and low feeding levels. A high between animal variation will increase the number of animals needed to document that treatments are significantly different.

In conclusion, chamber systems can be used to examine nearly all aspects of nutrition, and this technique gives results with a day-to-day CV, which can be below 10%, but the variation is dependent on e.g., feeding level. Considerations about design and placement of the chambers can eliminate the risk of reduced feed intake. There is no doubt that this system gives quantitative measurements of methane emission with low tolerance but establishment costs and limited capacity of the system restricts the number of animals, which can be examined experimentally.

## 3. Measuring Methane with the SF_6_ Tracer Technique

This method is relatively new and was first described in 1993–1994 [22,29]. The main purpose of the method was to investigate energy efficacy in free ranging cattle [29], because it had been queried that results obtained in respiration chambers could not be applied to free ranging animals [22,23]. The SF_6_ method is used widely in New Zealand [30,31], Canada [32,33], Australia [34,35] and the US [22,36], and also north European countries e.g., Sweden [37] and Norway [38] employ the method.

The basic idea behind the method is that methane emission can be measured if the emission rate of a tracer gas from the rumen is known. For this purpose a non-toxic [39], physiologically inert [40], stabile gas is needed. Furthermore, the gas should mix with rumen air in the same way as methane. SF_6_ was chosen [29], because it fulfills the above criteria, is cheap, has an extremely low detection limit and is simple to analyze.

**Figure 3:**
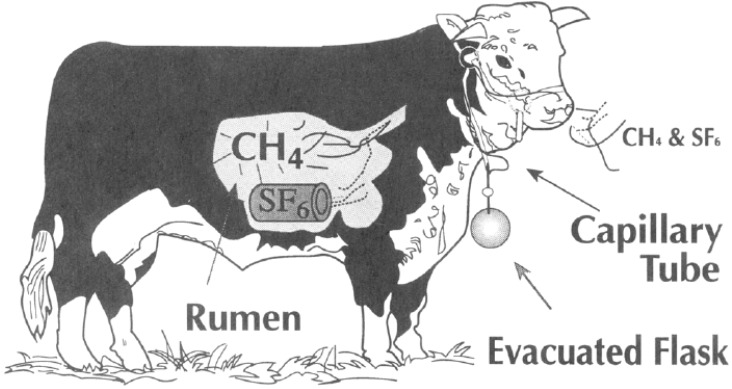
Illustration of the SF_6_ tracer technique. Reprinted with permission from [22]. Copyright (1994) American Chemical Society.

SF_6_ is filled into small permeation tubes. The rate of diffusion of SF_6_ out of the permeation tubes is measured by placing them in a 39 °C water bath and measuring the daily weight loss until it is stable. The permeation tube is then placed in the rumen of an experimental animal and collection of air can start. The sampling apparatus consists of a collection canister, a halter and capillary tubing. The capillary tubing is placed at the nose of the animal and connected with the evacuated canister (Figure 3). The tubing regulates the sampling rate. The sampling time is typically one day [22,29,41], but emission estimates from shorter time intervals have been published [30,36]. The concentration of SF_6_ and CH_4_ in the canister is determined by gas chromatography. For more detailed description of equipment and guidance see [41]. The methane emission is calculated from the release rate of SF_6_ and concentration of SF_6_ and CH_4_ in the containers in excess of background level [31] as described in Equation (1).

(1) $$F_{CH_{4}}=F_{tracer}\cdot \frac{C_{CH_{4}}^{measured}-C_{CH_{4}}^{atm}}{C_{tracer}^{measured}-C_{tracer}^{atm}}$$

where $F_{CH_{4}}$ is the total production of CH_4_, *F_tracer_* is the total production or release of SF_6_, $C_{CH_{4}}^{measured}$ and $C_{tracer}^{measured}$ are the measured concentrations of CH_4_ and SF_6_ in the experimental entity e.g., in the unit of ppm, while $C_{CH_{4}}^{atm}$ and $C_{tracer}^{atm}$ are the concentrations of CH_4_ and SF_6_ in atmospheric or background air, measured with the same analyzer and in the same unit.

Results based on direct measurements of gas composition in gas head space in the rumen of cannulated animals have also been published [36,42,43].

The system can be used to investigate nearly all aspects of feeding and nutrition e.g., level of feeding, effect of feedstuff, effect of chemical and physical composition, restricted *versus* *ad libitum* feeding, different additives and grazing. Using the method for investigation of dynamics of methane emission is debatable.

The method has been carefully tested during the last two decades and a number of difficulties have been described. The following problems will briefly be discussed: Maintaining a constant release rate from permeation tubes, effect of release rate upon emission rate of methane, background level determination, inconsistency between methane measurements determined in chambers and with SF_6_ and within and between animal variation.

The release rate is important and will affect emission estimates if not correctly determined. The release rate from permeation tubes is determined under laboratory conditions by weighing the permeation tubes regularly for at least 1½ months [41,44]. Only highly linear permeation tubes are used (R^2^ > 0.997) [45]. However, permeation curves have been shown to be slightly curvilinear under laboratory conditions [46,47]. Tests of permeation tubes pre- and post-experiments have also shown differences in permeation rate. Different methods to account for this are described by [46,47]. The permeation tubes are weighted in a laboratory in dry air and the release rate should be the same in the rumen. However, a 6–11% lower release rate in tubes placed in rumen fluid than in air has been observed [48].

It has also been shown that permeation tubes with high release rates give higher methane emissions than tubes with low release rates. It is therefore recommended to use permeation tubes with nearly the same release rate when comparison of different treatments is needed [48,49].

The measured concentration should be corrected for background levels of both SF_6_ and CH_4_ [31]. Measuring a representative background concentration under field condition can be difficult, because wind direction and other animals in the field can affect the concentrations [50].

Johnson *et al.* [22,51] observed a 7% lower methane emission with the SF_6_ technique than with chambers with cattle, and this can partly be explained by the few percent of methane, which is lost via rectum [52]. Comparisons [53,54] also showed a slightly lower emission (5–10%) with the SF_6_ technique than with chambers for both cattle and sheep. However, others have shown slightly higher values with the SF_6_ technique than chambers [26,55], and yet other studies have found much higher values with the SF_6_ technique than chambers [11,44,46,56,57].

Both within and between animal CV is much higher in experiments with the SF_6_ technique than with the chambers. In a study by Pinares-Patiño *et al.* [11] the same animals were measured both with the SF_6_ technique and in chambers. The within CV was 4.7, 13.5 and 11.7% in chambers, with SF_6_ and with SF_6_ in chambers, respectively. Also the between animal CV was twice as high with the SF_6_ technique as with the chambers. The correlation between the different methods is also inadequate. Both the higher within and between animal variations increase the number of measuring days and number of animals needed to verify differences between treatments.

In conclusion, the SF_6_ method gives more variable results of methane emission than chamber measurements. This increases the number of animals needed to prove treatment differences. The ability to use the method to quantify the methane emission has been debated in a number of studies [11,56], but the technique is still new and further investigation can hopefully improve the technique. The method is the only available method for measuring individual free ranging animals on e.g., pasture.

## 4. *In Vitro* Gas Production Technique for Methane Measurements

The *in vitro* gas production technique (IVGPT) has been used to simulate ruminal fermentation of feed and feedstuffs [58] for decades. With the increasing interest in green house gas (GHG) emissions from agriculture in recent years, the traditional IVGPTs have been modified to include measurement of methane production e.g., [59,60].

**Figure 4:**
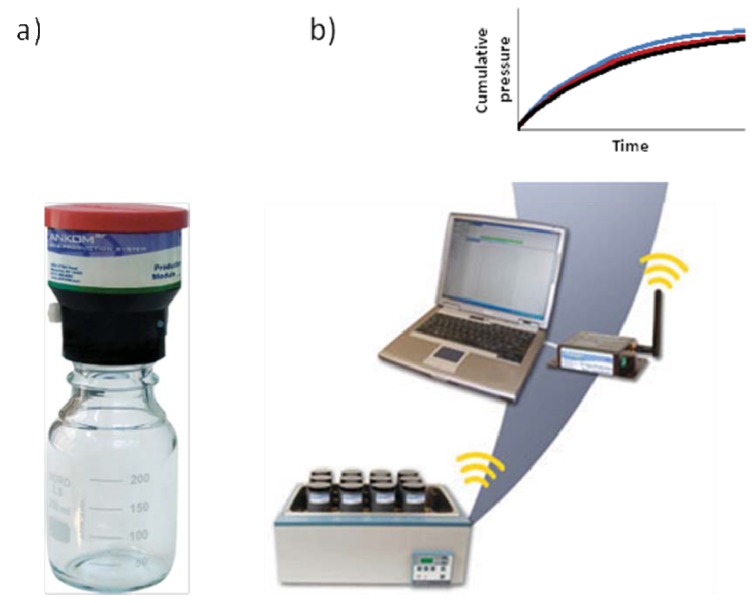
Illustration of a wireless *in vitro* gas production module. The individual gas production module (**a**) measures pressure from fermentation in the jar continuously and releases gas at a certain set point above atmospheric pressure. Data is wirelessly transferred from all modules, which can be incubated in a water bath or an incubator (**b**).

The basic principle of IVGPTs is to ferment feed under controlled laboratory conditions employing natural rumen microbes. Feedstuffs, e.g., subjected to different treatments, are incubated at 39 °C with a mixture of rumen fluid, buffer and minerals for a certain time period, typically 24, 48, 72, 96 or 144 h (Figure 4). The amount of total gas produced during incubation is measured and its composition analyzed, to obtain data on the *in vitro* production of methane. At the same time it is possible to determine *in vitro* degradation of the feedstuffs, making it possible to determine whether a reduction in methane production is at the cost of total feed degradation. The output of IVGPT experiments is usually reported as amount of CH_4_ per gram dry matter (DM), per gram degraded DM (dDM) or per gram degraded NDF (dNDF).

Various IVGPT systems have been employed for methane determination as for example syringes [61,62], rusitec [62], closed vessel batch fermentations [60] and lately fully automated systems [59]. Depending on the system and other laboratory constraints it is possible to conduct up to several hundred parallel incubations at a time, which allows for sufficient amounts of repetitions in experiments to support statistically significant differences between treatments. Residual variation between repeated measurements of methane production conducted with IVGPT is not well reported in the literature. A recent ring-test comparing *in vitro* gas production kinetics between laboratories reports a repeatabily (variation between duplicates within a series expressed as coefficient of variation (CV)) for asymptotic gas production ranging from 1.8% to 7.6% [63]. Similar or higher CV-percentages are therefore expected for *in vitro* methane determinations, where an extra analytical level has been added.

The typical time frame for conducting an *in vitro* experiment is 1–4 weeks, which makes it possible to screen many different feedstuffs and potential additives relatively fast and cheaply. The systems can also be used to explore dose-response curves for potential additives. Compared to *in vivo* experiments it is also much easier to control fermentation conditions like pH. The cow-to-cow variation observed *in vivo* can be avoided by using the same ruminal inoculum for all treatments which are to be compared *in vitro*. Preferably the inoculum is produced by mixing rumen fluid from several donor animals to include as many different rumen microbes as possible.

The method requires access to fresh rumen fluid, which is typically obtained from fistulated cows or other ruminants. Alternative methods of collecting rumen fluid are by esophageal tubing on intact animals or from slaughtered animals. The use of feces or cultures as alternative inoculants has been compared to fresh rumen fluid but only for total gas production/feed degradation [64]. Such alternative sources of inoculum are not expected to be applicable for estimation of CH_4_ production because of the complexity of the microbial ecosystem of the rumen.

Two studies comparing IVGPT measurements of CH_4_ production to the SF_6_-technique and the respiration chamber technique, respectively, show good agreement between the “whole animal”-techniques and IVGPT [62,65]. Results from other studies reporting both *in vitro* and *in vivo* give examples of both good agreement between methods as well as the opposite [66,67].

A clear disadvantage of IVGPT is that it only simulates the ruminal fermentation of feed, not emissions and digestibility by the entire animal. Furthermore, under normal conditions it does not include long-term adaptation of the ruminal microorganisms to the tested feedstuffs. It is common to use rumen fluid from animals on a standard feed ration. During live animal experiments it is common practice to have adaptation periods to new feeds of at least 14 days. The animals’ output is not considered stabile before that. For the methane-producing population of ruminal microbes there are indications that the adaptation period after switching to a new feed is more than 30 days [68].

IVGPT results should therefore always be interpreted with care, but it is a very useful technique e.g., as first approach to test potential feedstuffs and additives or when controlled incubation conditions are needed. The IVGP results can then be used to optimize larger and more expensive whole-animal experiments.

## 5. The CO_2_ Technique

A newly developed method for estimating methane emissions from livestock is based on the use of CO_2_ as a tracer gas [69]. Instead of using externally added SF_6_, the naturally emitted CO_2_ is used to quantify CH_4_ emission. The CH_4_/CO_2_-ratio in the production of air of the animal(s) in question is measured at regular intervals and combined with the calculated total daily CO_2_ production of the animal(s). The calculations are the same as for the SF_6_ tracer technique (Equation (1)), only with CO_2_ as the tracer gas instead of SF_6_.

The use of CO_2_ as a quantifier gas is based on knowledge compiled over more than 100 years from experiments measuring feed requirements and feed composition. The measured feed intake can be converted to heat-production, and there is a close relationship between heat- and CO_2_-production [70,71,72]. Animals at maintenance are thus emitting 1 L CO_2_ per 21.5–22.0 KJ of heat produced. Corrections can be made for lactating animals or animals gaining weight. The relation between heat production and CO_2_ production is partly related to the amount of fat deposited or mobilized and can in practice be as low as 20.0 KJ per L CO_2_ when large amounts of feed carbohydrates are converted to fat as in high yielding dairy cows. The total CO_2_ production from stables with different animals, e.g., lactating dairy cows, dry cows and heifers, has likewise been determined by researchers working with ventilation [73].

The CO_2_ method can be used to quantify methane production under different circumstances. Two examples are the total CH_4_ production from a whole stable with dairy cows [74] and individual estimates for cows visiting an automated milking system (AMS) [75]. A comparison with respiration chamber measurements has recently been published [76].

The expiration air of cattle contains CO_2_ and CH_4_ in concentrations 100 and 1000 times higher than the concentrations in atmospheric air, respectively. Therefore it is only necessary to have 5–10% of the animal’s breath in the air being analyzed. This can easily be achieved in a stable or when individual cows visit an AMS. The method can potentially be developed for application to grazing cattle. As about 95% of CH_4_ emissions from cows are excreted with expiration air [52], the small amounts excreted through the rectum can be ignored.

**Figure 5:**
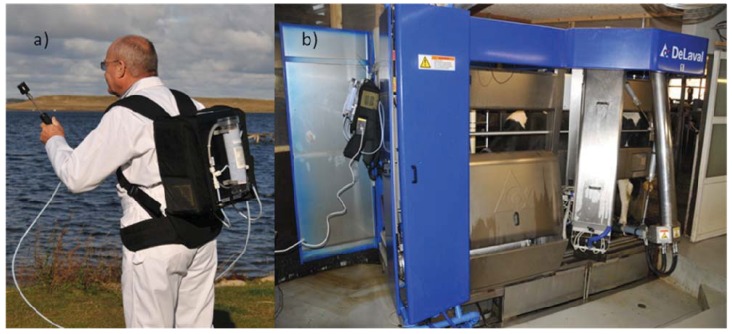
The portable Gasmet (**a**) used for measuring CH_4_ and CO_2_ concentrations in an automated milking system (**b**).

Measurements of CH_4_ and CO_2_ can be conducted with different types of analyzers - so far the CO_2_ method has used a portable equipment called Gasmet (Gasmet Tehcnologies Oy, Helsinki, Finland), which is based on infrared measurements (Fourier Transformed Infrared (FTIR), [77]) (Figure 5).

The equipment is portable and can easily be used under very different circumstances. The main disadvantage is that the CO_2_ production of animals is influenced by the same things as the animals’ requirement for energy. This means that the size, activity and production of the animal influences the amount of CO_2_ produced. This is not of importance when for instance the quantitative effect of different feeds or supplements on the methane production of different groups of equal animals is going to be established, but may produce larger errors when the quantitative methane production is going to be established on an individual animal or on different groups of animals. Combined with the only partial sampling of animal breath the estimation of individual animal emissions with the CO_2_-technique is expected to produce higher day-to-day variation than observed in respiration chambers. Fortunately, the method can easily be applied to many animals making it possible to reduce the standard error of means from experiments.

## 6. Other Measuring Techniques

### 6.1. Methods Based on Whole Buildings or Areas

The methods described to far are focused on single animal measurements that fit well within a traditional experimental agricultural setup and are well suited for comparing different treatments. Unfortunately, all these methods will affect animal behavior to some extent, and they are not suitable for measuring e.g., interactions between methane emission and barn design, exchange of methane between grazing animals and their surroundings or whole farm emissions.

During the last decades methods suitable for estimating methane emission both from barns, whole farms, feedlots and paddocks have been develop. For reviews see [3,78,79]. The methods can roughly be divided into non-micrometeorological techniques and micrometeorological techniques. Micrometeorological methods are defined as measuring fluxes of gas in the free atmosphere and relating these fluxes to animal emissions [78].

Two non-micrometeorological methods, which focus on systems rather than individual animals are described by [78]. The first is the external tracer ratio technique, where a tracer is released in the paddock or barn, and the concentrations of tracer and methane are measured in the surroundings [80,81,82]. The second technique is mass balance in barns, where ventilation rate and concentrations in inlet and outlet are used to estimate the emission [83].

The micrometeorological methods are based on measurements of wind velocity and methane concentration, but the number of measuring points and the theories used to calculate emission rates differ between methods. For further details of different technique see: Mass balance [80,84,85], Flux-gradient technique [84,86], Eddy covariance [87,88], Lagrangian dispersion analyses [89,90,91]. These methods are however influenced by instabilities like non-steady state wind or movement of point-emission sources. The recovery of released CH_4_ in a system tested by McGinn *et al.* [92] was measured to be 77%. It is also difficult to relate the CH_4_ production to feed intake for grazing animals, as most methods for assessing feed intake during grazing are associated with errors [93]. A recent study comparing a micrometeorological method with measurements in open-circuit respiration chambers does however show similar values of CH_4_ production per kg DMI [92].

The micrometeorological methods are still new and further development and documentation on reliability is needed, but the methods are valuable in evaluating whole dairy systems and interactions between animals and landscape.

### 6.2. Combined Feeder and CH_4_ Analyzer

A newly patented system called GreenFeedTM (C-lock Inc., USA) combines an automatic feeding system with measurements of CH_4_ and CO_2_. The animals entering an automatic feeding system are recognized and concentrations of CH_4_ and CO_2_ are measured. Air is continuously pumped through the automatic feeding system to quantify flow and thereby CH_4_ and CO_2_ emitted during eating. To ascertain how much of the expiration air is collected the system can perform recovery experiments automatically by releasing small amounts of a tracer gas inside the feeders head cabin. Possible applications are inside AMSs, in conventional tie-stalls, and for grazing animals fed supplements. A disadvantage is that it only measures methane emissions when the animals have their head in the feeder and are eating. Correlations with whole-day emissions must therefore be examined thoroughly.

### 6.3. Proxy Methods

Another type of method is being developed with the aim of examining many animals at a time without invasive intervention and large experimental set-ups. These so-called proxy-methods correlate methane emissions with parameters that can be measured in easily obtainable biological samples like milk or feces. Several studies have examined the fatty acid profiles of milk and correlated these with methane production of the animals. The theory is that certain fatty acids or fats in the milk or feces are correlated with either the feed composition [94] or the amount of methanogenic archae in the rumen [95], which both have an effect on the production of methane. Two recent studies [96,97] indicate some correlations between milk fatty acid profiles and methane emissions, but further studies are required.

## 7. Models for Predicting Methane Production 

In many cases measurements are just not possible e.g., when total national emissions have to be assessed. Therefore there is a global interest in being able to predict methane production using models based on existing data, such as animal characteristics (e.g., weight, breed), feed characteristics (e.g., nutrient and energy content), intake data (dry matter or nutrients) or digested nutrients. Such models often use data derived from experiments conducted with cattle in respiration chambers, but newer techniques for measuring methane, such as the SF_6_ or CO_2_ technique, have also been applied in recent years.

### 7.1. IPCC

Models for estimating cattle methane emissions have been developed which can be used to quantify the production of methane globally, nationally, or locally on the farm. The standard model usually used for calculating cattle methane emissions is issued by the IPCC (the Intergovernmental Panel on Climate Change). The IPCC, often also referred to as the “UN’s Climate Panel”, is an independent international scientific body established by the World Meteorological Organization and the United Nations Environment Programme (UNEP).

The IPCC’s most recent guidelines for estimating enteric methane emission are from 2006. The IPCC operates with three different levels to estimate greenhouse gas emissions [98]. These three levels depend on the quality of the database established in the country in question, and are known as Tiers 1, 2 and 3, where Tier 1 is the simplest calculation method and Tier 3 the most complex and data-dependent method. The three methods are based on the proportion of the cow’s gross energy intake (GE) excreted as methane. Tier 1 thus utilises an emission factor of 6.5% (Y_m_) and an assumed GE, which results in an estimated methane production of 109 kg/cow/year in Western Europe [98]. When using Tier 2, and especially Tier 3, more information is required to determine Y_m_, e.g., in relation to the digestibility and nutrient content of the feed. At the present time Y_m_ is mainly determined experimentally from measurements made in respiration chambers, but these will be updated as new techniques come into use. Models are also required to determine the feed and energy intake in relation to the cattle’s production in a given region/country. Furthermore, the use of an official Tier 3 method also requires scientific documentation via an article published in an international journal [98].

Countries participating in the Kyoto protocol submit an annual status report on their national GHG emissions to the UNFCCC (the United Nations Framework Convention on Climate Change). As standard, a CH_4_ emission coefficient of 6.5% of GE is used for cattle. Exempt from this are intensively fed cattle, defined as cattle receiving >90% concentrate, for which Y_m_ is determined to be 3% of GE [98].

As an example, the calculations of the Danish CH_4_ emissions employ an Y_m_ of 5.94% for dairy cattle instead of the standard emission coefficient of 6.5% of GE. This Danish factor is determined from calculations using the Karoline model [99], based on feed plans from approximately 15% of Danish dairy cows and the area of fodder beets in Denmark [100].

### 7.2. Methane Models 

Table 1 presents an overview of some recent methane models, which have been developed from measurements in respiration chambers. The table gives an impression of the considerable differences between the existing models in terms of the complexity of input required. Thus, the model developed in [21] only requires the proportion of roughage in the ration, while the model in [101] requires digested amounts of different nutrients. In practice, the latter model can thus only be used in conjunction with a digestion model such as that included in the NorFor feed evaluation system [102]. Several of the models in Table 1 have been developed on the basis of data containing information about the nutrient content of the feedstuffs. However, only few investigations have found that nutrient information improves the ability of the models to predict methane production [101], while e.g., [21] and [103] have found no such benefits of including nutrient information.

**Table 1:** Predictive methane equations developed from measurements in respiration chambers.

Reference	Equation	R^2^	N
IPCC [98] ^a^	Methane (kg/dag) = GE (MJ/d) × Y_m_/55.65		
Yan *et al.* [103] ^b^	Methane (L/d) = 47.8 × DMI − 0.76 × DMI^2^ − 41 (kg/d)	0.75	315
Yan *et al.* [103] ^bc^	Methane (L/d) = 0.34 × BW (kg) + 19.7 × DMI (kg/d) + 12	0.77	315
Kirchgessner *et al.* [104] ^d^	Methane (g/d) = 63 + 79 × CF + 10 × NFE + 26 × CP – 212 × Cfat (kg/d)	0.69	24
Jentsch *et al.* [101] ^de^	Methane (kJ/d) = 1.62 × d_CP − 0.38 × d_Cfat + 3.78 × d_CF + 1.49 × d_NFE +1142 (g/d)	0.90	337
Ellis *et al.* [21]	Methane (MJ/d) = 0.14 × forage (%) + 8.6	0.56	89
Mills *et al.* [105] ^f^	Methane (MJ/d) = 0.07 × ME (MJ/d) + 8.25	0.55	159
Mills *et al.* [105] ^b^	Methane (MJ/d) = 0.92 × DMI (kg/d) + 5.93	0.60	159
Mills *et al.* [105] ^b^	Methane (MJ/d) = 10.3 × forage (%) + 0.87 × DMI (kg/d) + 1.1	0.61	159
Grainger *et al.* [26] ^b^	Methane (g/d) = 18.5 × DMI (kg/d) − 9.5	0.56	16

^a^ GE = gross energy intake; Y_m_ = emission factor; ^b^ DMI = dry matter intake; ^c^ BW = body weight; ^d^ CF = crude fibre; NFE = N-free extract; CP = crude protein; Cfat = crude fat; ^e^ The equation is based on digested amounts which is designated with “d”; ^f^ ME = metabolizable energy intake.

A comparison of the above mentioned models leads to large differences in the estimates of methane emission, as was also found with a number of other models in a previous study [106]. The model estimates are also associated with error. The best equations developed by [21] for beef cattle, dairy cattle and beef+dairy cattle had prediction errors (RMSPE) of 14.4, 20.6 and 28.2%, respectively. The models described in [105] had RMSPE between 6.4% and 11.6% when evaluated with the dataset based on which they were developed, but when evaluated with independent datasets the RMSPE rose to 20.6% to 35.3%.

In the NorFor feed evaluation system, a wide range of nutrients consumed by the cow is calculated from a given ration, and the digested amounts of nutrients in the various digestive sections are available [102]. Work is underway by the Research and Development group of the NorFor co-operation to incorporate a predictive equation for methane into NorFor, whereby the methane emission will be estimated at ration level in connection with feed planning. This equation is being developed on the basis of recent methane studies undertaken by research institutions in Denmark, Norway and Sweden.

## 8. Comparison of Methods

It is thus evident, that several very different methods exist for measuring and estimating methane emissions from cattle. The best choice of method will very much depend on the purpose and the exact circumstances of each experiment. Table 2 contains some general parameters of the major methods described in this review.

**Table 2:** Comparison of different methods for measuring and estimating methane emissions from cattle.

Method parameters	Chambers	SF_6_ technique	*In vitro* gas production	CO_2_ technique	IPCC	Other models
Prerequisites (except for instruments)			Access to rumen fluid	Information about CO_2_ production. Can be calculated [70].	Information about e.g., number of animals, intake of gross energy	Model dependent, e.g., dry matter intake, nutrient composition
*Aspects of feeding which can be investigated*						
Feeding level	Yes	Yes	No	Yes	No	Yes—some models
Physical form of the feed	Yes	Yes	No (all feed is ground)	Yes	No	No
Chemical composition of diet	Yes	Yes	Yes	Yes	No	Yes—some models
Supplementation of feed additives	Yes	Yes	Yes	Yes	No	No
*Influence on animals*						
Fixation needed	Yes	No	*	Depends on aim	*	*
Animal needs to carry equipment	No	Yes	*	Depends on aim	*	*
Can be used in milking parlor or automatic milking	No	No	*	Yes	*	*
*Method estimates*						
Individual animals	Yes	Yes	No	Yes	Yes	Yes
Within animal variation	Yes	Yes	No	Yes	No	No
Between animal variation	Yes	Yes	No	Yes	No	No
Daily variation	Yes	No	No	Yes	No	No
Time resolution ^1^	A few minutes to hours	8–24 h	Min. 6 h	Small intervals of a few minutes	*	*
*Output format*						
Basic	l CH_4_/day/animal	l CH_4_/day/animal	l CH_4_/kg dry matter	l CH_4_/day/animal	l CH_4_/day/animal	l CH_4_/day/animal
Relative to dry matter intake	Yes	Yes	Yes	Yes	Yes	Yes
Relative to digested organic matter	Yes	Yes	Yes	Yes	No	Yes, depends on model
Relative to digested NDF	Yes	Yes	Yes	Yes	No	Yes
Relative to milk yield	Yes	Yes	No	Yes	*	Yes
Relative to gross energy intake	Yes	Yes	No	Yes	*	Yes

* Not relevant for the method; ^1^ Will depend on individual system settings.

The chamber method has good accuracy and precision for assessing the daily production of CH_4_ from housed animals but limited capacity with regards to the number of animals. It is therefore best suited for comparison of distinct mitigation strategies in crossover or Latin square experiments. It also provides information on the daily variation in methane emission. It is not applicable to free ranging animals, and because of the limited capacity and the need for training of animals, it cannot be recommended for screening animals, e.g., for assessment of heredity of methane emission.

The SF_6_ technique has the clear advantage of being suitable for free ranging animals, which constitute a large proportion of livestock worldwide. Like the chamber method it gives good estimates of the between animal and within animal variation in emissions and is also well suited for comparison of distinct mitigation strategies. The residual variation on measurements is higher than with the chamber method, but it is possible to employ more animals per experiment, which provides a better statistical foundation for testing hypotheses. The animals need to carry equipment around and must therefore be selected and trained before the experiments start.

Screening large amounts of feeds and additives is the best application of the *in vitro* method. This method has a large capacity, making it possible to test many different combinations of feedstuffs and e.g., doses of additives. It is therefore well suited for initial screening of potential treatments before *in vivo* experiments are initiated. The physical, chemical and microbial environment can be kept more constant between *in vitro* repeats (within runs) than between individual animals in chamber or SF_6_ experiments. However, the method only simulates ruminal fermentation and cannot take into account physical factors like passage rate of digesta or physical form of the feed

The CO_2_ technique is a newly developed approach to estimation of methane emissions from cattle. It can be used under different conditions e.g., estimation of the heredity of methane emission potential by individual measurements on large amounts of animals or for the overall estimation of herd-level emissions from a barn. To estimate total CH_4_ emission this method relies on calculated values of total CO_2_ production. It will therefore be less precise than the chamber methods for estimating individual animal emissions. For comparison of mitigation strategies this can however be compensated by increasing the number of animals.

Other methods like the micrometeorological and the proxy techniques are also on the way and may prove very useful in the future.

Finally, the mathematical models are essential for estimating national or global emissions. They are easy to apply and will give estimates of the average emission of the unit(s) in question. The models are based on experimental data and as such are limited in their application by the limitations on the experimental data. A model based on respiration chamber experiments can therefore not be directly applied to free ranging cattle. Furthermore, our understanding of ruminal digestion is not complete, so neither are the models of it. Therefore a continuous need exists for more data to increase our knowledge of this complex system.

## 9. Conclusions

Many good methods for measuring and estimating methane emissions from ruminants are already in use and new ones are being developed. None of them are however flawless and they all require careful consideration before application. In this context a thorough knowledge of the advantages and disadvantages of experimental methods is extremely important, both when planning experiments and when interpreting own results and the published findings of other researchers.
